# The Association Between Clinical Nurses’ Work Environment, Job Stress, and Health Locus of Control and Presenteeism in South Korea

**DOI:** 10.3390/healthcare12222293

**Published:** 2024-11-16

**Authors:** Jin-Young Park, Yong-Sook Eo

**Affiliations:** College of Nursing, Dongguk University-WISE, Gyeongju 38066, Republic of Korea; peridot_0817@naver.com

**Keywords:** work environment, job stress, health locus of control, presenteeism, clinical nurses

## Abstract

Background: This study aimed to examine the relationship between the work environment, job stress, and health locus of control and presenteeism among clinical nurses in South Korea. Methods: A cross-sectional, descriptive correlational study was conducted on clinical nurses (n = 276) from general hospitals in two small cities. Data were collected via a self-administered structured questionnaire from 1 to 14 December 2022. Descriptive and stepwise multiple regression analyses were conducted for this study. Results: The work environment (ß = −0.28, *p* < 0.001) and education (ß = −0.13, *p* = 0.031) were significant predictors of health problems, explaining 10% of the variance. Job stress (ß = 0.34, *p* < 0.001), external locus of control (ß = 0.25, *p* < 0.001), physician control locus (ß = −0.15, *p* = 0.006), work department (ß = −0.13, *p* = 0.018), and marital status (ß = −0.13, *p* = 0.022) significantly predicted job impairment, accounting for 25% of the variance. For perceived productivity, job stress (ß = −0.18, *p* = 0.003), marital status (ß = 0.18, *p* = 0.002), and external locus of control (ß = −0.16, *p* = 0.007) were influential, explaining 8% of the variance. Conclusions: To mitigate presenteeism among clinical nurses, interventions should focus on improving the work environment at the organizational level and addressing job stress and health locus of control at the individual level. By implementing targeted strategies, healthcare institutions can reduce job impairment and enhance productivity among nursing staff.

## 1. Introduction

Many scholars and practitioners have noted that presenteeism causes significant costs in the workplace, leading to active discussions worldwide [1]. Presenteeism refers to the phenomenon where workers come to work despite having health issues [2], resulting in a loss of productivity [3]. These losses reportedly account for about 77% of organizational productivity decline [4]. In the United States, the costs associated with presenteeism, estimated at 180 billion, surpass those of absenteeism, which total 118 billion [5]. Unlike absenteeism, presenteeism is less visible since employees continue to report to work, making it a critical issue to address [6].

In the nursing profession, individuals are particularly susceptible to physical, environmental, and psychological stressors, increasing their risk of health complications. Interestingly, the rate of absenteeism among nurses is not significantly higher than that in other professions [7]. Many Korean nurses continue to work despite experiencing chronic or minor health issues, rather than taking time off [8]. This phenomenon contributes to reports of understaffing, excessive workloads, and a strong sense of responsibility among nurses, all exacerbated by presenteeism [9,10,11]. In the healthcare sector, nurses encounter significant organizational and social pressures to maintain attendance [11]. Despite feeling overwhelmed, they persist until tasks are completed [12]. Presenteeism in clinical nurses diminishes job commitment, adversely affecting the quality of nursing services and patient satisfaction and ultimately impacting job performance and organizational productivity [13]. Thus, addressing presenteeism in clinical nurses is vital not only for their health but also for organizational efficiency, requiring comprehensive strategies.

Johns proposed a model to understand presenteeism by examining it at both individual and organizational levels [3]. According to his model, work-related, personal, and health-related factors interact dynamically to contribute to presenteeism. Work-related factors include contextual elements like understaffing, heavy workloads, and job responsibility. Personal factors encompass attitudes toward work, personality, and sex. These factors, combined with the nature of the illness (acute, episodic, or chronic), influence whether an employee will exhibit presenteeism or absenteeism [3]. This model underscores the goal of managing presenteeism to enhance employee health and boost organizational productivity.

Within the context of clinical nursing, the work environment emerges as a critical work-related factor. A supportive work environment is essential for delivering high-quality nursing care [14] and is integral to organizational productivity. Conversely, a detrimental work environment is often linked to a greater incidence of health issues [15], which can diminish the quality and productivity of nursing [16]. Prior studies have indicated that nurses frequently encounter unfavorable working conditions characterized by inadequate benefits, low wages, and insufficient organizational support [17]. Nevertheless, many nurses choose to attend work while ill in order to meet performance expectations [18].

Job stress has been identified as a major influence on presenteeism among clinical nurses [8,10]. Nurses experience job stress due to societal demands for high-quality care, interpersonal conflicts, job insecurity, and workplace culture [8,10]. Excessive job stress diminishes productivity and efficiency, complicating the delivery of quality care and the management of patient care [8]. High levels of job stress are closely associated with health problems and job impairment, making it a significant factor in presenteeism [10].

Health locus of control, a personal factor, refers to an individual’s beliefs about what influences their health [19]. Individuals with a strong internal health locus of control believe their health outcomes are determined by their own decisions, efforts, and actions more than external forces such as powerful others, fate, or luck [20]. Those with strong internal beliefs, such as self-efficacy and health locus of control, are more likely to seek and utilize personal health resources when making health-related decisions [21]. A previous study examining the relationship between health locus of control and presenteeism among university students found that a higher health locus of control was associated with lower levels of sick presenteeism [22]. However, those with a strong internal health locus of control who find it difficult to refuse requests may be more inclined to work even when ill [3,23]. Personality factors have been integrated into models for understanding presenteeism [24], indicating that the nature of the illness (acute, episodic, chronic) should be considered when examining presenteeism [25]. Therefore, a comprehensive understanding of presenteeism among clinical nurses also necessitates consideration of health locus of control as a personality factor.

This study aims to identify the relationships among the work environment, job stress, and health locus of control with respect to presenteeism among clinical nurses. Given the current lack of emphasis on health locus of control as a relevant factor in presenteeism specifically among clinical nurses, the study seeks to investigate these relationships by incorporating health locus of control alongside existing situational work factors and job stress.

## 2. Materials and Methods

### 2.1. Study Design and Participants

This study employed a cross-sectional, descriptive correlational design to examine the relationship among clinical nurses’ work environment, job stress, health locus of control, and presenteeism. Participants were clinical nurses from two general hospitals in cities with populations under 500,000. Eligibility criteria included having worked for over one year, understanding the study’s purpose, and voluntarily providing written consent. The sample size was calculated using G*Power 3.1.9.7, assuming a two-tailed significance level (α), power (1 − β) = 0.95, and a medium effect size (d) = 0.15 with 15 predictor variables in a regression analysis. The minimum required number of participants was determined to be 199. To account for possible dropouts, 290 questionnaires were distributed initially. After excluding 14 incomplete or erroneously filled questionnaires, 276 participants were included in the final analysis. To ensure the ethical protection of participants, this study received approval from the Institutional Review Board (IRB) of the university with which the researchers are affiliated (IRB No.: DGU IRB 20220029).

### 2.2. Measures

Data were collected through self-report questionnaires that assessed the participants’ general characteristics, work environment, job stress, health locus of control, and presenteeism. General characteristics encompassed nine items: sex, age, marital status, education level, work experience, work type, job position, department, and night shifts.

#### 2.2.1. Work Environment

The work environment was assessed using an adapted version of the 6th Korean Working Conditions Survey by the Korean Occupational Safety and Health Research Institute [26], which is grounded in the dynamic model of work context factors proposed by Johns [3]. The revised questionnaire included 10 items covering motivation, teamwork, career prospects, job sustainability, compensation adequacy, job demands, substitution ease, organizational climate, absenteeism policy, and job flexibility. Content validity was established through evaluation by a researcher experienced in instrument development for clinical nurses and 11 nurse managers with over 20 years of clinical experience and at least a master’s degree. The item-level content validity index (I-CVI) ranged from 0.91 to 1.00, while the scale-level content validity index (S-CVI) was 0.96, exceeding the criteria of I-CVI ≥ 0.78 and S-CVI ≥ 0.90 when evaluated by six or more experts [27,28]. Additionally, face validity was confirmed with 30 general nurses who had more than one year of experience, yielding an I-CVI = 0.91–1.00 and an S-CVI = 0.94. The reliability of the instrument was established with a Cronbach’s α of 0.80.

#### 2.2.2. Job Stress

Job stress was measured using the short-form version of the Korean Occupational Stress Scale, originally developed by Chang et al. [29] and subsequently abbreviated by Jung, Lee, Arakida [30]. This scale comprises seven sub-dimensions: job demands (4 items), job autonomy (4 items), interpersonal conflict (2 items), job insecurity (3 items), organizational system (3 items), inadequate compensation (5 items), and workplace culture (3 items). Each item was rated on a 4-point Likert scale, with higher scores indicating increased job stress. In Jung et al.’s [30] study, the tool demonstrated a reliability of Cronbach’s α = 0.81, while in this study, the reliability was Cronbach’s α = 0.84.

#### 2.2.3. Health Locus of Control

Health locus of control was measured using the Multidimensional Health Locus of Control Scale (MHLC) developed by Wallston et al. [19] and adapted into Korean by Shin and Kang [31]. The tool contains 18 items divided into four sub-scales: internal health locus of control (6 items), health locus of control relying on others (3 items), doctor health locus of control (3 items), and chance health locus of control (6 items). Each item was rated on a 5-point Likert scale, with higher scores indicating a stronger corresponding locus of control. In Shin and Kang’s [31] study, the tool’s reliability was Cronbach’s α = 0.70 for internal control, 0.61 for reliance on others, 0.66 for doctor control, and 0.77 for chance control. In this study, the corresponding Cronbach’s α values were 0.64, 0.67, 0.51, and 0.70, respectively.

#### 2.2.4. Presenteeism

Presenteeism was assessed using the Stanford Presenteeism Scale (SPS), developed by Turpin et al. [32] and translated by Jung et al. [30]. This instrument measures three sub-factors: health issues, work impairment, and perceived productivity. Health issues encompass 14 health conditions experienced in the past month, such as allergies, arthritis, asthma, back pain, respiratory issues, depression, insomnia, diabetes, hyperlipidemia, liver dysfunction, cardiovascular problems, headaches, gastrointestinal disorders, and others. Participants reported whether they were currently experiencing any of these conditions, with the total score reflecting the number of health issues identified (ranging from 0 to 14). They were also asked to specify the health problem of greatest concern. Work impairment was measured using 10 items on a 5-point Likert scale, with higher scores indicating greater impairment. The reliability of this sub-scale was Cronbach’s α = 0.83 in Turpin et al. [32], 0.80 in Jung et al. [30], and 0.76 in this study. Perceived productivity was Jung et al. [30], and 0.76 in this study. Perceived productivity was measured using a visual analog scale ranging from 0% to 100%, with higher percentages indicating greater perceived productivity despite health issues.

### 2.3. Data Collection

Data collection occurred from 1 to 14 December 2022. The researchers visited the nursing departments of two general hospitals in the cities, explained the study’s purpose and data collection process to department heads, and obtained permission to proceed. Both verbal and written explanations of the study were provided to the nurses, highlighting its purpose, guarantee of anonymity, voluntary nature of participation, right to withdraw at any time, and potential benefits and risks. After obtaining written informed consent, the nurses completed the questionnaires. Each questionnaire was placed in an envelope, sealed, and immediately collected by the researcher. Participants received a small token of appreciation upon completion. All responses were then entered into a database, and the completed questionnaires were securely stored and later safely destroyed to prevent any personal information leakage.

### 2.4. Data Analysis

The collected data were coded and analyzed using IBM SPSS (version 26, Armonk, NY, USA). Descriptive statistics such as frequency, percentage, mean, and standard deviation were employed to analyze the general characteristics of the participants. Mean and standard deviation were also utilized to examine the participants’ work environment, job stress, health locus of control, and presenteeism. Differences in the work environment, job stress, health locus of control, and presenteeism based on general characteristics were analyzed using t-tests and ANOVA, with Scheffé post hoc tests conducted to further explore significant differences identified in the ANOVA results. Given that these variables were measured on interval or ratio scales, Pearson correlation coefficients were employed to analyze the relationships among them. The decision to use Pearson’s correlation was based on the assumption that the data met the necessary criteria of normality and homoscedasticity. As such, prior to performing the analysis, Shapiro–Wilk tests were conducted to assess normality, and Levene’s test was used to evaluate the equality of variances. In addition to correlation analysis, stepwise regression analysis, incorporating both forward and backward selection methods, was executed to identify significant factors influencing the outcome variables of interest [33]. This approach not only elucidated the relationships among variables but also facilitated a deeper understanding of the predictors of job stress and presenteeism, thereby strengthening the overall analysis.

## 3. Results

### 3.1. General Characteristics of the Participants

Regarding the participants’ general characteristics, 9.4% were male, and 90.6% were female. The average age was 31.16 ± 7.89 years, with the majority (58.4%) being under 30 years old. In terms of marital status, 71.9% were unmarried, and 28.1% were married. Regarding education, 83.9% held a bachelor’s degree or higher, while 16.1% had an associate degree. The average clinical experience was 7.97 ± 7.83 years, with 52.4% having less than 5 years of experience. Another 27.3% had over 10 years of experience, and 20.2% had between 5 to 10 years. Concerning work shifts, 85.0% worked shifts, while 15.0% worked standard hours. Most participants (82.4%) were staff nurses, while 17.6% held supervisory roles. Work departments included general wards for 64.4% of participants and specialized units for 35.6%. Additionally, 82.8% of participants worked night shifts (Table 1).

### 3.2. Work Environment, Job Stress, Health Locus of Control, Presenteeism Level, and Health Issues

The average score for the work environment was 3.01 ± 0.39, and for job stress, it was 2.46 ± 0.31. Among the sub-factors of health locus of control, physician-related control scored the highest at 3.15 ± 0.64, while chance-related control scored the lowest at 2.30 ± 0.60. Presenteeism was assessed by sub-factors: health problems scored 2.37 ± 1.52, job impairment scored 2.53 ± 0.52, and perceived productivity was 76.93 ± 15.06. Of the participants, 80.5% reported having 1–3 health issues, with 48.4% reporting chronic problems and 43.4% reporting temporary problems (Table 2).

The most common health issues reported were shoulder stiffness and back pain (51.3%), followed by headaches (36.0%), a tendency toward insomnia (35.6%), and digestive issues (35.2%). The health issues most frequently treated were allergies (6%), shoulder stiffness and back pain (4.5%), and digestive issues (3%). The health problems of greatest concern to participants were shoulder stiffness and back pain (22.8%), a tendency toward insomnia (15.7%), and digestive issues (15%) (Table 3).

### 3.3. Differences in Presenteeism Based on General Characteristics

Differences in presenteeism based on general characteristics are detailed in Table 4. Significant differences in health problems, a sub-factor of presenteeism, were observed based on education level (t = 2.91, *p* = 0.004) and years of experience (F = 5.09, *p* = 0.007). Job impairment also showed significant differences based on marital status (t = 2.28, *p* = 0.023) and work department (t = −3.25, *p* = 0.001). Perceived productivity was significantly affected by sex (t = 2.24, *p* = 0.031), marital status (t = −3.02, *p* = 0.003), and work type (t = −2.03, *p* = 0.046).

### 3.4. Correlation Between Work Environment, Job Stress, Health Locus of Control, and Presenteeism

The participants’ work environment was negatively correlated with health problems (r = −0.30, *p* < 0.001) and job impairment (r = −0.26, *p* < 0.001), both sub-factors of presenteeism. Conversely, it showed a positive correlation with perceived productivity (r = 0.16, *p* = 0.011). Job stress was positively correlated with health problems (r = 0.28, *p* < 0.001) and job impairment (r = 0.39, *p* < 0.001) but negatively correlated with perceived productivity (r = −0.19, *p* = 0.002). Among the sub-factors of health locus of control, chance control exhibited a negative correlation with health problems (r = −0.13, *p* = 0.037) and a positive correlation with job impairment (r = 0.20, *p* = 0.001), along with a negative correlation with perceived productivity (r = −0.17, *p* = 0.004). External locus of control showed a positive correlation with job impairment (r = 0.26, *p* < 0.001) and a negative correlation with perceived productivity (r = −0.17, *p* = 0.006). Physician control was negatively correlated with job impairment (r = −0.13, *p* = 0.036) (Table 5).

### 3.5. Predictors of Presenteeism

The variables included in the stepwise regression analysis were checked for multicollinearity issues. The tolerance values ranged from 0.658 to 0.973, all above 0.1, and the variance inflation factor (VIF) values were between 1.027 and 1.519, well below the threshold of 10, indicating no risk of multicollinearity. The Durbin–Watson statistic ranged from 1.869 to 2.026, close to 2, suggesting no autocorrelation in the residuals. Therefore, the assumptions of the regression model were met, validating the interpretation of the results.

To identify significant predictors of the health problems sub-factor of presenteeism, education level and years of experience, which showed significance in the general characteristics, were included as independent variables. Education was treated as a dummy variable. Additionally, work environment, job stress, and chance control, which were significant in the correlation analysis, were also included. The results indicated that the work environment (ß = −0.28, *p* < *0*.001) and education (ß = −0.13, *p* = 0.031) were significant predictors, explaining 10% of the variance (F = 16.061, *p* < 0.001).

For the job impairment sub-factor of presenteeism, marital status and work department, significant in the general characteristics, were treated as dummy variables. The analysis also included work environment, job stress, and health locus of control (chance, others, and physician) as independent variables. The results demonstrated that job stress (ß = 0.34, *p* < 0.001), control by others (ß = 0.25, *p* < 0.001), control by physicians (ß = −0.15, *p* = 0.006), work department (ß = −0.13, *p* = 0.018), and marital status (ß = −0.13, *p* = 0.022) were significant, with an explanatory power of 25% (F = 18.353, *p* < 0.001).

For the perceived productivity sub-factor of presenteeism, sex, marital status, and work department, significant in the general characteristics, were treated as dummy variables. Additionally, work environment, job stress, and health locus of control (chance and others) were included as independent variables. The analysis indicated that job stress (ß = −0.18, *p* = 0.003), marital status (ß = 0.18, *p* = 0.002), and control by others (ß = −0.16, *p* = 0.007) were significant predictors, with an explanatory power of 8% (F = 8.826, *p* < 0.001) (Table 6).

## 4. Discussion

This study found that the level of presenteeism, particularly related to health issues, averaged 2.37 out of a possible 14 points, with every nurse (100%) reporting at least one health problem. This finding is consistent with previous studies indicating that a significant proportion, 93.6% [34] and 97.0% [12], of clinical nurses experience one or more health issues. The most prevalent health complaint was musculoskeletal pain, particularly in the shoulders and back, which aligns with findings from both domestic [10,34] and international studies, including a Japanese study where 76.7% of clinical nurses identified musculoskeletal pain as their primary health concern [35]. Musculoskeletal disorders among clinical nurses are often linked to prolonged standing, excessive force exertion, and repetitive tasks associated with patient care and transportation [34]. Therefore, it is critical to thoroughly assess the physical factors contributing to musculoskeletal issues in nurses and implement preventative guidelines as well as ergonomically designed protocols.

Chronic health issues were reported by 48.4% of the nurses, with work impairment due to chronic conditions ranging from 17.8% to 36.4%, increasing with the number of chronic illnesses [36]. In this study, 24.4% of nurses were receiving treatment for recognized health problems, which is similar to the 25.2% reported by Lee and Lee [37] but higher than the 19.3% noted by Ko et al. [34]. Regarding musculoskeletal pain, 4.5% were undergoing treatment, consistent with previous findings [34,37]. These results indicate that many nurses continue to work despite health issues without actively seeking treatment. Specifically, only 1.1% of those experiencing insomnia and 0.4% of those with mental health issues such as depression and anxiety were receiving treatment, despite 35.6% and 14.2% respectively reporting such conditions. This underscores the need for heightened attention to mental health, as nurses often endure high emotional labor, which can precipitate depression [38]. Currently, the Korean healthcare system mandates annual health check-ups for nurses; however, there is a lack of systematic follow-up management for identified health issues [34]. Consequently, hospitals need to develop comprehensive health management systems that regularly evaluate and address both the physical and emotional health needs of nurses.

Several demographic factors were identified as predictors of presenteeism. Education level was associated with health issues, while marital status and work department were linked to job impairment, and marital status also influenced perceived productivity. Nurses with lower education levels reported more health issues, while unmarried nurses experienced higher job impairment and lower perceived productivity compared to their married counterparts. Nurses working in general wards also faced greater job impairment than those in specialized wards. The correlation between education level and presenteeism corroborates findings by Yeom et al. [39]. The relationship between marital status and presenteeism aligns with studies by Ko et al. [36] and Kwon and Kim [40], which focused on industrial nurses. Although marriage often entails dual responsibilities of work and childcare [8], recent policies aimed at balancing work and family life, such as family-friendly management and flexible work hours, may have influenced these results [41]. It is important to continue supporting married nurses through family-friendly policies and to identify factors contributing to job impairment among unmarried nurses. Further investigation is warranted, as suggested by discrepancies with findings from Lee et al. [42]. Work environment, job stress, and health locus of control were confirmed as major predictors of presenteeism. Specifically, the work environment had a significant relationship with health problems, a key sub-factor of presenteeism. Poor work environments correlated with increased health issues, consistent with previous studies [15,43]. A supportive nursing work environment not only enhances patient care but also positively affects nurse retention and job satisfaction [44]. In the U.S., fewer than 20% of nurses consider their work environment safe, with unsafe conditions contributing to stress, fatigue, and both acute and chronic illnesses [45]. Similarly, Korean nurses contend with inadequate welfare systems, low pay, and insufficient institutional support, all of which adversely affect their health [17]. Alnuhait et al. [46] reported that nurses have the highest levels of presenteeism among occupational groups, largely due to time pressures and heavy workloads. The self-sacrificing identity common among nurses can lead to negative health outcomes [35], and many continue working despite health issues, often due to difficulties in adjusting schedules or a reluctance to burden colleagues [12]. Managers should therefore strive to create work environments that enhance accessibility to job resources.

Job stress was identified as a crucial predictor of job impairment and perceived productivity. It was the most significant predictor of job impairment, aligning with findings from previous studies [8,40]. Perceived productivity, which reflects the level of labor exerted despite health problems [32], tends to decline when employees work while ill [43]. Job stress occurs when demands are high and personal control is low [47], and for nurses, it often stems from shift work, long hours, role conflicts, and excessive workloads. These conditions are linked to health issues such as gastrointestinal disorders and musculoskeletal symptoms [48], which directly affect perceived productivity. Addressing job stress through targeted interventions is essential to reduce presenteeism effectively.

Health locus of control was also a predictor of job impairment and perceived productivity. A higher external health locus of control, particularly belief in the influence of others, was associated with increased job impairment, while a lower external health locus of control correlated with higher perceived productivity. Health locus of control refers to a person’s beliefs about what controls their health [19], with a higher internal health locus of control typically associated with better health behaviors [49]. Furthermore, a study involving university students found that a higher health locus of control is linked to lower levels of sick presenteeism [22]. However, no significant relationship between internal health locus of control and presenteeism was found among clinical nurses. In contrast, an association was found between external health locus of control and presenteeism, which differs from the findings of studies conducted among university students. Johns [24] asserted that individuals with a high internal health locus of control are more likely to attend work despite experiencing pain, highlighting the importance of recognizing personal characteristics, such as health locus of control, in relation to presenteeism among employees. Furthermore, research indicates that individuals with personalities that struggle to refuse requests from others are more inclined to attend work when ill [23]. These findings suggest that challenging work environments—exacerbated by difficulties in schedule adjustments due to staffing shortages [12]—may compel clinical nurses to work while unwell, resulting in higher job impairment and lower productivity.

The “physician health control” factor was noted as contributing to lower job impairment. In studies related to patient care, individuals who believed their health was determined by authoritative figures, such as physicians and healthcare professionals, demonstrated higher adherence to self-care practices [50]. This finding suggests that nurses highly value the judgments of professionals, particularly physicians, within the context of external health locus of control. Consequently, they may be less likely to engage in presenteeism when their illness is directly assessed by a physician. However, due to the low reliability of the “physician health control” tool, caution is advised when interpreting these results. Given the limited research directly linking health locus of control with presenteeism, further studies are needed to enhance our understanding of this relationship.

This study has several limitations. First, the sample was drawn from clinical nurses employed in general hospitals across two cities, which may limit the generalizability of the results due to potential sampling bias. Second, while the study identified relationships among the work environment, job stress, health locus of control, and presenteeism, the explanatory power was relatively low, and the cross-sectional nature of the design restricts the ability to establish causal relationships. Finally, there is a scarcity of prior research specifically linking the work environment and health locus of control with presenteeism, complicating the interpretation of the findings. Future research should further explore these constructs, including assessments of their validity and reliability, to better inform interventions aimed at reducing presenteeism among clinical nurses.

## 5. Conclusions

This study integrated both work environment factors and personal factors, including job stress and health locus of control, to identify the predictors of presenteeism among clinical nurses. The findings indicated that a poor work environment and lower education levels were associated with increased health problems. Additionally, higher job stress, an external health locus of control, marital status, and assignment to general wards were linked to greater job impairment. Conversely, reduced job stress and lower external health locus of control were associated with higher perceived productivity. Therefore, to manage presenteeism effectively among clinical nurses, it is important to adopt multifaceted approaches. These strategies should focus on improving the work environment, reducing job stress, and addressing personal health perceptions. Enhancements to physical working—including staffing levels and salary systems—along with the creation of a supportive work atmosphere that encourages nurses to report health issues and take necessary time off, are crucial. Such measures are important for reducing job impairment and increasing overall productivity.

## Figures and Tables

**Table 1:** Participants’ characteristics (n = 267).

Characteristics	Categories	n (%)	M ± SD
Sex	Male	25 (9.4)	
	Female	242 (90.6)	
Age (year)	<30	156 (58.4)	31.16 ± 7.89
	30~<40	67 (25.1)	
	≥40	44 (16.5)	
Marital status	Not married	192 (71.9)	
	Married	75 (28.1)	
Education level	Diploma	43 (16.1)	
	Bachelor or higher	224 (83.9)	
Total clinical career (year)	<5	140 (52.4)	7.97 ± 7.83
	5~<10	54 (20.2)	
	≥10	73 (27.3)	
Work shift	Shift work	227 (85.0)	
	Non-shift work	40 (15.0)	
Job position	Staff nurse	220 (82.4)	
	Charge nurse or higher	47 (17.6)	
Work department	Special unit	95 (35.6)	
	General unit	172 (64.4)	

M, mean; SD = standard deviation.

**Table 2:** Participants’ work condition, job stress, health locus of control, and presenteeism (n = 267).

Variables	Categories	n (%) or M ± SD	Min, Max	Range
Work environment	-	3.01 ± 0.39	2.0–4.30	1–5
Job stress	-	2.46 ± 0.31	1.75–3.54	1–4
Health locus of control	Internal control	2.91 ± 0.54	1.0–4.33	1–5
	Others’ control	2.40 ± 0.71	1.0–4.33	1–5
	Physician control	3.15 ± 0.64	1.33–4.67	1–5
	Chance control	2.30 ± 0.60	1.0–4.17	1–5
Presenteeism	Health problem	2.37 ± 1.52	1.0–8.0	1–14
	Health problem frequency			
	1~3	215 (80.5)		
	4~6	48 (18.0)		
	≥7	4 (1.5)		
	Health problem type			
	Acute	20 (8.2)		
	Intermittent	106 (43.4)		
	Chronic (≥6 month)	118 (48.4)		
	Job impairment	2.53 ± 0.52	1.0–4.3	1–5
	Perceived productivity	76.93 ± 15.06	20–100	0–100

M, mean; SD = standard deviation.

**Table 3:** Participants’ health problem types (n = 267).

Health Problem Types	* Currently Experienced	* Currently Being Treated	Most Concerning
	n (%)	n (%)	n (%)
Allergies	37 (13.9)	16 (6.0)	12 (4.5)
Arthritis	50 (18.7)	6 (2.2)	16 (6.0)
Asthma	7 (2.6)	3 (1.1)	1 (0.4)
Shoulder/back pain	137 (51.3)	12 (4.5)	61 (22.8)
Respiratory issues	4 (1.5)	0 (0.0)	1 (0.4)
Depression/anxiety	38 (14.2)	1 (0.4)	10 (3.7)
Insomnia	95 (35.6)	3 (1.1)	42 (15.7)
Diabetes	3 (1.1)	2 (0.7)	2 (0.7)
Hyperlipidemia	17 (6.4)	2 (0.7)	6 (2.2)
Liver dysfunction	6 (2.2)	2 (0.7)	2 (0.7)
Cardiovascular issues	14 (5.2)	2 (0.7)	10 (3.7)
Headaches	96 (36.0)	3 (1.1)	33 (12.4)
Digestive issues	94 (35.2)	8 (3.0)	40 (15.0)
Other	11 (4.1)	6 (2.2)	8 (3.0)
Allergies	37 (13.9)	16 (6.0)	12 (4.5)
Arthritis	50 (18.7)	6 (2.2)	16 (6.0)
Asthma	7 (2.6)	3 (1.1)	1 (0.4)
Shoulder/back pain	137 (51.3)	12 (4.5)	61 (22.8)

* Multiple responses allowed.

**Table 4:** Differences in presenteeism based on participant characteristics (n = 267).

Characteristics	Categories	Presenteeism					
		Health Problem		Job Impairment		Perceived Productivity	
		M ± SD	t or F (*p*)	M ± SD	t or F (*p*)	M ± SD	t or F (*p*)
Sex	Male	2.08 ± 1.58	−0.99 (0.332)	2.37 ± 0.61	−1.65 (0.099)	81.20 ± 9.27	2.24 (0.031)
	Female	2.40 ± 1.51		2.55 ± 0.51		76.49 ± 15.48	
Age	<30	2.19 ± 1.41	2.88 (0.058)	2.53 ± 0.55	0.67 (0.514)	76.25 ± 14.53	2.76 (0.065)
	30~<40	2.55 ± 1.64		2.58 ± 0.50		75.37 ± 17.95	
	≥40	2.72 ± 1.61		2.46 ± 0.46		81.70 ± 10.89	
Marital status	Not married	2.29 ± 1.56	−1.39 (0.166)	2.58 ± 0.54	2.28 (0.023)	75.34 ± 15.53	−3.02 (0.003)
	Married	2.57 ± 1.40		2.42 ± 0.46		81.00 ± 13.02	
Educational attainment	Diploma	2.98 ± 1.82	2.91 (0.004)	2.56 ± 0.51	0.42 (0.672)	78.02 ± 15.47	0.52 (0.604)
	Bachelor or higher	2.25 ± 1.43		2.53 ± 0.53		76.72 ± 15.01	
Work experiences (years)	<5	2.23 ± 1.54 ^a^	5.09 (0.007)	2.51 ± 0.53	0.87 (0.419)	75.64 ± 14.60	1.12 (0.329)
	5–<10	2.09 ± 1.28 ^b^		2.61 ± 0.53		78.80 ± 15.81	
	10+	2.84 ± 1.55 ^c^		2.52 ± 0.50		78.01 ± 15.34	
	Scheffé		a < c				
Work type	Shift work	2.33 ± 1.53	−1.05 (0.294)	2.52 ± 0.54	−0.70 (0.486)	76.28 ± 15.48	−2.03 (0.046)
	Non-shift work	2.60 ± 1.48		2.59 ± 0.42		80.63 ± 11.89	
Position	Staff nurse	2.34 ± 1.54	−0.71 (0.476)	2.54 ± 0.54	0.31 (0.760)	76.30 ± 15.48	−1.49 (0.137)
	Charge nurse or higher	2.51 ± 1.43		2.51 ± 0.42		79.89 ± 12.62	
Work department	Special unit	2.14 ± 1.57	−1.85 (0.066)	2.39 ± 0.50	−3.25 (0.001)	78.00 ± 13.79	0.86 (0.389)
	General unit	2.49 ± 1.48		2.61 ± 0.52		76.34 ± 15.73	
Night shift	Non	2.52 ± 1.44	0.76 (0.449)	2.54 ± 0.43	0.17 (0.868)	80.11 ± 11.86	1.89 (0.063)
	Yes	2.33 ± 1.54		2.53 ± 0.54		76.27 ± 15.59	

M = mean; SD = standard deviation. ^a, b, c^ Groups with the same letter are not significant different from each other (Scheffé test, *p* < 0.05)

**Table 5:** Relationship between work environment, job stress, health locus of control, and presenteeism (n = 267).

Variables	Categories	WE	JS	HLC				P		
				ILC	CLC	OLC	PLC	HP	JI	PP
		r (*p*)	r (*p*)	r (*p*)	r (*p*)	r (*p*)	r (*p*)	r (*p*)	r (*p*)	r (*p*)
WE		1								
JS		−0.58 (<0.001)	1							
HLC	ILC	0.27 (<0.001)	−0.21 (0.001)	1						
	CLC	0.17 (0.006)	0.01 (0.856)	0.36 (<0.001)	1					
	OLC	0.03 (0.665)	0.11 (0.077)	0.36 (<0.001)	0.62 (<0.001)	1				
	PLC	0.06 (0.371)	−0.03 (0.676)	0.21 (0.001)	0.10 (0.092)	0.22 (<0.001)	1			
P	HP	−0.30 (<0.001)	0.28 (<0.001)	−0.07 (0.271)	−0.13 (0.037)	−0.03 (0.630)	−0.07 (0.292)	1		
	JI	−0.26 (<0.001)	0.39 (<0.001)	−0.09 (0.145)	0.20 (0.001)	0.26 (<0.001)	−0.13 (0.036)	0.18 (0.004)	1	
	PP	0.16 (0.011)	−0.19 (0.002)	0.06 (0.351)	−0.17 (0.004)	−0.17 (0.006)	0.06 (0.294)	−0.12 (0.043)	−0.40 (<0.001)	1

WE = work environment; JS = job stress; HLC = health locus of control; ILC = internal locus of control; CLC = chance locus of control; OLC = others locus of control; PLC = physician locus of control; P = presenteeism; HP = health problem; JI = job impairment; PP = perceived productivity.

**Table 6:** Factors influencing presenteeism (n = 267).

Variable	Presenteeism														
	Health Problem					Job Impairment					Perceived Productivity				
	B	SE	*ß*	t	*p*	B	SE	*ß*	t	*p*	B	SE	*ß*	t	*p*
(Constant)	5.92	0.69		8.59	<0.001	1.14	0.28		4.13	<0.001	104.66	7.50		13.96	<0.001
WE	−1.09	0.23	−0.28	−4.79	<0.001										
JS						0.58	0.09	0.34	6.32	<0.001	−8.63	2.90	−0.18	−2.98	0.003
OLC						0.19	0.04	0.25	4.60	<0.001	−3.43	1.26	−0.16	−2.72	0.007
PLC						−0.13	0.05	−0.15	−2.77	0.006					
Marital status (ref. = unmarried)						−0.15	0.06	−0.13	−2.31	0.022	6.11	1.97	0.18	3.10	0.002
Educational attainment (ref. = diploma)	−0.53	0.24	−0.13	−2.17	0.031										
Work department (ref. = general ward)						−0.14	0.06	−0.13	−2.37	0.018					
	R^2^ = 0.11, Adj.R^2^ = 0.10, F = 16.061, *p* < 0.001					R^2^ = 0.26, Adj.R^2^ = 0.25, F = 18.353, *p* < 0.001					R^2^ = 0.09, Adj.R^2^ = 0.08, F = 8.826, *p* < 0.001				

WE = work environment; JS = job stress; OLC = others locus of control; PLC = physician locus of control.

## Data Availability

Data are available upon request due to ethical and privacy restrictions.
